# Differences in fruit and vegetable intake and their determinants among 11-year-old schoolchildren between 2003 and 2009

**DOI:** 10.1186/1479-5868-8-141

**Published:** 2011-12-22

**Authors:** Claudia Fischer, Johannes Brug, Nannah I Tak, Agneta Yngve, Saskia J te Velde

**Affiliations:** 1 The EMGO+ Institute for Health and Care Research and the Department of Epidemiology and Biostatistics, VU University Medical Center, Van der Boechorststraat 7, 1081 BT, Amsterdam, The Netherlands; 2 Department of Health Sciences, VU University, De Boelelaan 1081, 1081 HV, Amsterdam, The Netherlands; 3 Department of Biosciences and Nutrition, Karolinska Institutet, Novum, SE-141 83, Huddinge, Sweden; 4 Department of Health, Nutrition and Management, Oslo and Akershus University College, P.O.Box 4, St Olavsplass, NO0130 Oslo, Norway

**Keywords:** schoolchildren, fruit and vegetables, trend, the Netherlands

## Abstract

**Background:**

Fruit and vegetable (FV) intake in children in the Netherlands is much lower than recommended. Recurrent appraisal of intake levels is important for detecting changes in intake over time and to inform future interventions and policies. The aim of the present study was to investigate differences in fruit and vegetable intake, and whether these could be explained by differences in potential determinants of FV intake in 11-year-old Dutch schoolchildren, by comparing two school samples assessed in 2003 and 2009.

**Methods:**

For 1105 children of the Pro Children study in 2003 and 577 children of the Pro Greens study in 2009 complete data on intake and behavioural determinants were available. The self-administered questionnaire included questions on children's ethnicity, usual fruit and vegetable intake, mother's educational level, and important potential determinants of fruit and vegetable intake.

Multiple regression analysis was applied to test for differences in intake and determinants between study samples. Mediation analyses were used to investigate whether the potential mediators explained the differences in intake between the two samples.

**Results:**

In 2009, more children complied with the World Health Organization recommendation of 400 g fruit and vegetables per day (17.0%) than in 2003 (11.8%, p = 0.004). Fruit consumption was significantly higher in the sample of 2009 than in the sample of 2003 (difference = 23.8 (95%CI: 8.1; 39.5) grams/day). This difference was mainly explained by a difference in the parental demand regarding their child's intake (23.6%), followed by the child's knowledge of the fruit recommendation (14.2%) and parental facilitation of consumption (18.5%). Vegetable intake was lower in the 2009 sample than in the 2003 sample (12.3 (95%CI -21.0; -3.6). This difference could not be explained by the assessed mediators.

**Conclusions:**

The findings indicate that fruit intake among 11-year-olds improved somewhat between 2003 and 2009. Vegetable intake, however, appears to have declined somewhat between 2003 and 2009. Since a better knowledge of the recommendation, parental demand and facilitation explained most of the observed fruit consumption difference, future interventions may specifically address these potential mediators. Further, the provision of vegetables in the school setting should be considered in order to increase children's vegetable intake.

## Introduction

A healthy diet, including an ample intake of fruit and vegetables, is part of recommendations for a healthy diet and may be especially important in youth [1]. Fruit and vegetables supply part of the important nutrients needed for physical and mental development during childhood [2]. This is the time when food and meal habits are learned [3], which tend to track to a certain extent into adulthood [4]. Furthermore, ample intakes of fruit and vegetables are believed to contribute to prevention of chronic disease [5,6] and possibly weight management [7].

The World Health Organization (WHO) and Food and Agriculture Organization (FAO) recommend a daily intake of 400 grams of fruit and vegetables [8]. The Dutch recommendation for fruit and vegetable intake for 10-12-year-old-children is at least two pieces of fruit and 150-200 g of vegetables per day [9] Many schoolchildren fall short of the recommended amount per day [9], however, and a cross-country comparison indicated that Dutch 11-year-old children have lower intakes than children in many other European countries [10].

Similarly to other countries in Europe [11,12] and elsewhere [13,14], government and non-governmental health promotion agencies in the Netherlands have included fruit and vegetable promotion in health promotion efforts targeting the population in general and schoolchildren in particular. For this reason several initiatives have been undertaken in the Netherlands in the last few years to gain more insight into the intake pattern and to increase the consumption of fruit and vegetables among children. Campaigns like the 'Vita+Froet' project [15], the Schoolgruiten Project [16] and the Pro Children Project [17,18] focused on interventions in the school setting, because the advantage of this setting is that almost all children can be reached.

A main focus of such projects is increasing the availability and accessibility of fruit and vegetables at school by providing children with free servings of fruit and/or ready-to-eat vegetables during school hours. Some also use specific school lessons to increase knowledge, for example, or promote taste testing to enhance preferences, and parental activities to improve parental support [15,17]. Some municipal health services in the Netherlands have adopted the *Schoolgruiten *programme; there are initiatives to implement the programme nationwide, and the European Commission is encouraging similar promotion of fruit and vegetable consumption across Europe [19].

To explore trends in intake, inform decisions about continuation of the aforementioned interventions or develop future programmes and policies, it is important to gain insight into present intake levels and correlates of intakes as compared with the period before most intervention activities were launched [20].

Therefore, the objectives of the present study were to explore differences in the fruit and vegetable consumption and their determinants between two national representative samples of 11-year-old schoolchildren in the Netherlands in 2003 and 2009. In addition, it was assessed whether differences in the presumed determinants explained the potential differences in intake levels between the two samples. Finally, it was tested whether differences between the samples were modified by gender, ethnic background or parental educational level.

## Methods

### Sample

Representative samples of 11-year-olds in the Netherlands were derived from the Dutch cross-sectional survey data of the Pro Children Study in 2003 [21] and the data of the Dutch survey conducted within the Pro Greens study in 2009 [22]. Both studies were funded by the European Commission and used the same sampling and survey methodology.

### Procedures

The methodological build-up was identical in both studies and has been described in more detail previously [21]. The data collection process of the Pro Children study took place between October and November 2003; the Pro Greens study collected data between April and June 2009. Supervised by the teachers, children completed a self-administered written questionnaire in one school hour. Another questionnaire was given to the children to take home for their parents. Parental informed consent was obtained before the children participated in the surveys [17]. Ethical approval for Dutch participation in the Pro Children study was obtained by the Medical Ethics Committee of the Erasmus University Medical Centre Rotterdam; Dutch participation in the Pro Greens study protocol was approved by the Medical Ethics Committee of the VU University Medical Center, Amsterdam.

### Measures

#### Fruit and vegetable intake

Primary outcome measures were the total intake of fruit and vegetables in grams on the day prior to the day of the data collection. Fruit and vegetable intakes were analysed separately because previous studies have shown that these are distinct behaviours [23] with their own determinants and may be affected differently by interventions. Fruit and vegetable intake was measured by a validated self-administered 24-hour recall questionnaire [24].

In summary, the 24 h recall is an instrument that identifies all vegetables and fruits consumed during the day prior to the completion of the questionnaire. Questions were asked about the fruit and vegetable intake in three different time intervals: (1) before school, (2) during school time and lunch, (3) after school, at supper and after supper. Amounts were indicated in terms of the number of slices, portions, or pieces eaten and standards were defined for these units [24]. Dried fruit and fruit juice were not included in the assessment of fruit intake; qualitative research shows that soft drinks, lemonades or fruit yogurts are often regarded as fruit juice by children. Potatoes were not included in the vegetable intake assessment [24]. Over-reporters, defined as reporting more than 1000 gram/day of total fruit and vegetable intake (excluding fruit juices) [24], were excluded from the analyses (10 children, five in each sample). Both studies used the same questionnaire, but with one exception: the questionnaire of the Pro Greens study had an extra item on berry consumption, which was covered in the 'other fruit' section in the Pro Children study.

For descriptive purposes only, intake levels were dichotomized according to whether children met the WHO recommended daily amount of fruit and vegetables of 400 grams/day and whether the children met the Dutch recommendations (at least two pieces of fruit per day; at least 150 grams of vegetables per day).

#### Potential determinants/mediators

Potential mediators of interest were previously identified determinants of fruit and/or vegetable intake. These potential mediators were assessed for fruit and vegetable intake separately by a questionnaire that had previously shown acceptable to good test-retest reliability (12 out of 15 fruit and vegetable questions had an intra-class correlation coefficient (ICC) > .0.60) [25]. A range of important potential mediators of fruit intake as well as vegetable intake were chosen based on a previously published theoretical framework [21]. The included variables were personal factors (knowledge of the daily fruit and vegetable intake recommendation - 'knowledge fruit', 'knowledge vegetables', general liking of fruit and vegetables - 'liking'), perceived environmental factors (availability of fruit and vegetables at school - 'school availability'), social factors, i.e. active parental encouragement to eat fruit and vegetables - 'active encouragement', whether parents facilitate intake of fruit and vegetables by cutting them up for their child - 'facilitating', whether parents demand their child eats fruit and vegetables -'parental demand' and whether parents allow their child to eat as much fruit and vegetables as they want to - parental allowing'. The exact formulation of the questions and the response alternatives are presented in Table 1. All items were assessed with a bipolar five-point scale ranging from fully disagree (-2) to fully agree (+2), higher values reflecting more positive beliefs regarding a high fruit or vegetable intake. 'Knowledge fruit', 'knowledge vegetables' and 'school availability' were dichotomized (see Table 1). When constructs ('liking', 'active encouragement') were assessed by two or more items the mean of the items was taken.

**Table 1:** Exact description of the mediators used in the study and test-retest reliability, if available

Constructs with items	Response categories	Test-retest reliability (ICC)
**PERSONAL**		
**Liking**		
I like to eat fruit/vegetables every day	5-point scale from 2 = 1 I fully agree to -2 = I fully disagree	Fruit: ICC = 0.74
Fruit/vegetables taste good		Vegetable: ICC = 0.77
**Knowledge fruit**		
How much fruit do you think you should eat to have a healthy diet?	1 = no fruit, 2 = 1-3 pieces per week, 3 = 4-6 pieces per week, 4 = 1 piece per day, 5 = 2 pieces per day, 6 = 3 pieces per day, 7 = 4 pieces per day, 8 = 5 pieces per day or more; Recoded: correct knowledge = (5-8) = 1, incorrect (1-5) = 0	ICC = 0.52

**Knowledge vegetables**		
How many vegetables do you think you should eat to have a healthy diet?	1 = no vegetables, 2 = 1-3 portions (serving spoons) per week, 3 = 4-6 portions per week, 4 = 1 portion every day, 5 = 2 portions every day, 6 = 3 portions every day, 7 = 4 portions every day, 8 = 5 or more portions every day; Recoded: correct knowledge = 6-8	ICC = 0.61

**Perceived social environmental**		
**Active encouragement**		
My mother encourages me to eat fruit/vegetables every day	5-point scale from 2 = 1 I fully agree to -2 = I fully disagree	Fruit: ICC = 0.73
My father encourages me to eat fruit/vegetables every day		Vegetable: ICC = 0.64

**Parental demand**		
Do your parents demand that you eat fruit/vegetables every day?	5-point scale from 2 = yes, always to -2 = never	Fruit: ICC = 0.68
		Vegetable: ICC = 0.71

**Parental allowing**		
Are you allowed to eat as much fruit/vegetables as you like at home?	5-point scale from 2 = yes, always to -2 = never	Fruit: ICC = 0.50 Vegetable: ICC = 0.59

**Facilitating**		
Does your mother or father usually cut up fruit/vegetables for you in between meals?	5-point scale from 2 = yes, always to -2 = never	

**Perceived physical environment**		
**School availability**		
Can you get fruit/vegetables at school either by buying it or getting it for free?	5-point scale from 2 = yes, always to -2 = never	
**Bringing to school**		
Do you usually bring fruit/vegetables with you to school?	5-point scale from 2 = yes, always to -2 = never	

#### Potential confounders and effect modifiers

Age, gender, ethnic background of the children and their mother's educational level were assessed as potential confounders or effect modifiers. Mother's educational level was categorized according to years of education: fewer than 12 years and 12 and more years.

Children's ethnic background was defined in three categories according to Statistics Netherlands: native Dutch (both parents were born in the Netherlands), non-Western (at least one of the parents was born in a non-Western country) and western ethnic background (at least one of the parents was born outside the Netherlands but in Europe (excluding Turkey), North America, Oceania, Japan, or Indonesia). Children's ethnic background, gender, and age as well as their mother's educational level were respectively obtained from the child and parent questionnaires.

### Respondents and preliminary data handling

**Figure 1:**
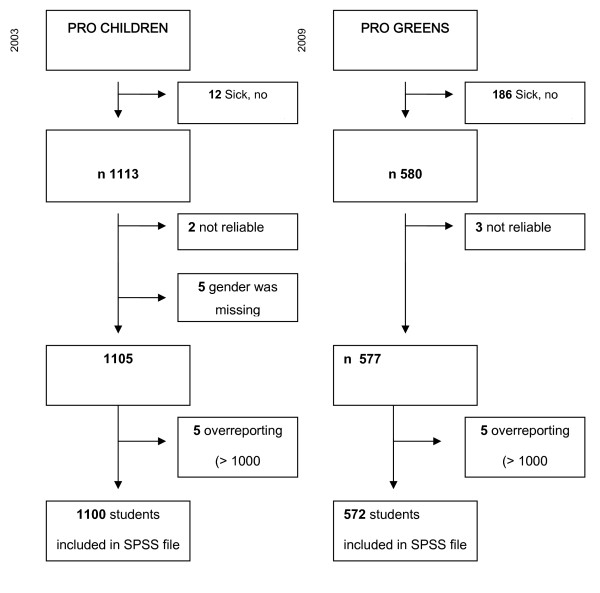
**Flow diagram of the inclusion process of the Pro Children study and the Pro Greens study**.

As can be seen in the flow diagram (Figure 1), a total of 1891 pupils (1125 children from the Pro Children study and 766 children from the Pro Greens study) were eligible to participate in the study. As Figure 1 shows, a number of children were excluded for several reasons, which resulted in a total of 1682 children (1105 children from the Pro Children study and 577 subjects from the Pro Greens study). For these children 883 parents' data files were available in the Pro Children study and 557 in the Pro Greens study. Ten children, however, were additionally excluded from the analyses because they were identified as over-reporters owing to their intake being more than 1000 gram/day. This resulted in 1100 children from the Pro Children study and 572 children from the Pro Greens study providing complete data on fruit and/or vegetable intake.

### Statistical analysis

Descriptive statistics were calculated for the key variables. Student's t test, the chi-square test or the non-parametric Mann-Whitney U test were applied to compare crude differences in intake levels and potential determinants between the two study samples.

The 24 h recall data showed a considerably skewed distribution, because many children reported that they had not eaten fruit or vegetables on the day prior to the survey and thus many zeros were scored. A log transformation (ln(x+1)) did not result in a normal distribution. Therefore, analyses for all outcome variables were carried out on non-transformed data. The distributions of the residuals from the regression analysis were checked for all analyses and found to be acceptable. Since the 'allowing' variables were highly skewed to the right, they were categorized as 'negative', i.e. taking 'strongly disagree' and 'disagree' together; 'neutral', and 'positive', i.e. taking 'strongly agree' and 'agree' together. Likewise, the 'bringing vegetables to school' variable was dichotomized into a low (including the two negative answer alternatives) and a 'neutral/high' category, including the neutral and two positive answer categories.

All analyses with fruit and vegetable intake and potential determinants as dependent variables and the two samples (coded zero and one) as an independent variable were conducted with linear or binary logistic regression analyses. First, effect modification by age, gender, ethnic background of the child and/or the mother's educational level was assessed by including the interaction terms between the group variable and the potential moderator. Children's age, gender, mother's educational level and ethnic background of the child were included as covariates in the adjusted models to account for potential confounding.

Second, it was explored whether the differences in presumed determinants could explain the differences in intake between the samples (the so-called 'total effect' or path c). Mediation analyses were applied in accordance with MacKinnon *et al. *[26] and following the SPSS script developed by Preacher and Hayes [27]. Briefly, the association between sample and presumed determinants (path a) and the association between the presumed determinants and the intake variables (path b) were assessed. The product-of-coefficient method [26] was then applied to calculate the mediated effects (a*b). Bootstrapping with 5000 re-samples was used to construct the bias-corrected 95% confidence intervals around the mediated effects [27]. The proportion of the 'total effect' explained by the presumed determinants was calculated as a*b/c for each individual determinant and as Σ(a_i_*b_i_)/c for all determinants together. For both fruit intake and vegetable intake two mediation models were run; the first model including all presumed mediators and a second model including only the variables that were identified as significant mediators in the first model.

All analyses were conducted with the PASW Statistics 18 program (IBM). A p-value ≤ 0.05 was considered to be significant. Numbers included in the specific analyses differ slightly because of missing values on one or more variables.

## Results

### Characteristics

As shown in Table 2 the children in the Pro Children study were slightly older than the children in the Pro Greens study. In both studies, there were slightly more girls than boys. In both samples the majority of the children were of native Dutch background, but there were more children of Western ethnic background in the Pro Children study. Educational level was high in both samples. More children in the Pro Greens sample met the WHO recommendation of at least 400 g of fruit and vegetables than in the Pro Children sample. With regard to the Dutch recommendations, however, the children from the Pro Children sample performed better, especially on the recommendation for fruit intake.

**Table 2:** Characteristics of the study population

		Total population N = 1672				
			
		Pro Children N = 1100		Pro Greens N = 572		
		**N**	**Mean (SD) or %**	**N**	**Mean (SD) or %**	P-value*

age (years)		1096	12.1 (5.9)	572	11.6 (5.2)	0.065
gender (boys)		509	46.3%	272	47.6%	0.642
ethnic background of child						
	Non-Western	113	10.3%	45	7.9%	
	Western	69	6.3%	18	3.2%	= 0.005
	Native Dutch	918	83.5%	508	89.0%	

educational level of the mother						
	< 12 years	250	28.5%	180	32.9%	= 0.085
	≥ 12 years	628	71.5%	367	67.1%	

Meeting the recommendation WHO of 400 g fruit and vegetables per day		130	11.8%	97	17.0%	= 0.004

Meeting Dutch fruit intake recommendation (at least 2 pieces/day)		748	68%	226	39.5%	< 0.001

Meeting the Dutch vegetable intake recommendation (at least 150 gram/day)		133	12.1%	58	10.1%	= 0.257

fruit intake (gram/day)						
girls	Mean (SD)	591	141.3 (132.5)	300	159.5 (168.8)	= 0.105
	Median (IQR)		100 (50; 200)		100 (0; 200)	
boys	Mean (SD)	509	124.8 (135.5)	272	150.3 (162.0)	= 0.027
	Median (IQR)		100 (0; 200)		100 (0; 200)	
Vegetable intake (gram/day)						
		
girls	Mean (SD)	591	72.7 (83.6)	300	64.1 (83.6)	0.137
		
	Median (IQR)		60 (0; 100)		50 (0; 100)	
		
boys	Mean (SD)	509	67.5 (80.6)	272	67.5 (80.6)	0.036
		
	Median (IQR)		60 (0; 117.5)		30 (0; 60)	

SD- standard deviation; IQR - Interquartile range, p25-p75

* as estimated by X^2 ^test (independent categorical data) or t-test for independent samples (for continuous data)

### Fruit and vegetable intake

As can be seen in Table 3 the difference in fruit intake between the two samples was 23.8 gram/day (95%CI 6.7; 36.2) in favour of the children from the Pro Greens sample. This effect was not modified by gender, age, origin or maternal educational level.

**Table 3:** Regression coefficients (β) and 95% confidence intervals (95% CI) as results of multiple linear regression analyses for differences in fruit and vegetable intake between children of the Pro Children and the Pro Greens samples

	Fruit intake (grams/day)		
	
	β	95%CI	
Model 1	21.5	6.7;	36.2
Model 2	23.8	8.1;	39.5
	**Vegetable intake (grams/day)**		
	
	**β**	**95%CI**	
Model 1	-12.7	-21.5;	-4.0
Model 2	-12.3	-21.0;	-3.6

Model 1 - crude analysis; Model 2 - adjusted for age, adjusted for children's ethnic background, + adjusted for mother's educational level

β - reflects difference between the fruit/vegetable intake of children in the Pro Children sample (0) and Pro Greens sample (1)

Table 3 shows that the 2009 sample had a significantly lower vegetable intake than the 2003 sample. The difference in mean vegetable intake between the two samples was small: (-12.7 (-21.5; -4.0) gram/day) in the crude and (-12.3 (-21.0; -3.6) gram/day) in the adjusted analyses. No significant effect modification was observed (data not shown).

### Potential determinants of fruit and vegetable intake

Table 4 shows the unadjusted differences in the scores between the two samples on the potential determinants for fruit and vegetable intake. For fruit intake, the samples differed in almost all presumed determinants, except for liking (but there was a trend towards statistical significance, p = 0.073) and parental encouragement. More children of the Pro Greens sample knew the recommendation for fruit intake; they scored higher on the demand rule; however fewer children reported a high level of allowance to eat fruit, but they reported more facilitation by their parents. Finally, the children from the Pro Greens sample reported bringing fruit to school more often and reported a somewhat higher school availability, although it was still low. Differences between the two samples were also observed for the presumed determinants of vegetable intake: children of the Pro Children sample more often knew the recommendation for vegetable intake; children from the Pro Greens sample scored higher on the demand rule; however they reported lower levels of allowance. Finally, the Pro Greens sample reported more availability at school, but perceived availability of vegetables was still poor (only 2.1%).

**Table 4:** Median scores and interquartile ranges (IQR) for determinants of fruit and vegetable intake

	Pro Children (PC)			Pro Greens (PG)			
**FRUIT INTAKE**	**N**	**Median**	**IQR (p25-p75)**	**N**	**Median**	**IQR (p25-p75)**	P*

knowledge fruit (% of children who scored correctly)	1101	48%		581	59.7%		< 0.001
liking (-2; +2)	1101	1.5	1 - 2	584	1.5	1 - 2	0.073
active encouragement (-2; +2)	1097	0	-1 - 1	580	0.5	-1 - 1	0.691
parental demand(-2;+2)	1090	0	0 - 1	574	1	0 - 2	< 0.001
parental allowing	1084			559			= 0.022
Neutral	110	10.1%		68	12.2		
High	920	84.9%		447	80.0%		
facilitating (-2; +2)	1090	0	-1 - 1	581	0	0 - 1	< 0.001
bringing fruit to school (-2; +2)	1100	-1	-2 - 0	580	0	-1 - 1	< 0.001
school availability (% of children scoring positive)	1086	1.3%	576		4.0%		= 0.001

**VEGETABLE INTAKE**							
Knowledge vegetables (% of children who scored correctly)	1100		22%	583	19%		< 0.001
liking (-2; +2)	1100	1	0 - 1.5	582	1	0 - 1.5	0.708
active encouragement (-2; +2)	1096	1	0 - 1.5	578	1	0 - 1.5	0.537
parental demand(-2;+2)	1095	1	0 - 1	580	1	0 - 2	0.003 (PC < PG)
parental allowing	1093			570			0.006
Neutral	146	13.4%		97	17.0%		
High	888	81.2%		426	74.7		
facilitating (-2; +2)	1085	-1	-2 - 0	574	-1	-2 - 0	0.718
bringing vegetables to school	1088			559			0.415
Low	937	86.7%		473	84.6%		
Neutral/high	151	13.9%		86	15.4%		
school availability (% of children scoring positive)	1086	0.7%		563	2.1%		0.018

* p-value based on Mann-Whitney U test or X^2 ^test between the Pro Children sample and the Pro-Greens sample

* IQR- interquartile range, p25 - p75

As can be seen from Table 5 the mediation analyses showed that the difference in fruit intake could mainly be explained by three variables: knowledge of the Dutch fruit intake recommendation (14.2%), parental demand (23.6%) and parental facilitation (18.5%). For the other variables there was no significant association with either the sample (path a) or with the outcome variable (path b).

**Table 5:** Results from the mediation analyses exploring whether presumed determinants could explain differences in fruit and vegetable intake between the two samples (0 = Pro Children; 1 = Pro Greens)

fruit intake (n = 1369)	total effect = 25.16 (7.36; 43.0)						direct effect model 1 = 13.0 (-4.44; 30.5)				Direct effect in model 2 = 10.0 (-5.63; 25,7)			
	path a		path b (model 1)		path b (model 2)		mediated effect (a*b) in model 1				mediated effect (a*b) in model 2			
	coeff	SE	coeff	SE	coeff	SE	coeff	95%CI^1^		proportion	coeff	95%CI^1^		proportion
knowledge	**0.13**	0.05	**25.30**	7.82	**26.47**	7.86	**3.28**	**1.28**	**6.44**	13.0%	**3.27**	**1.24**	**5.99**	14.2%
liking	0.04	0.08	**35.24**	4.92	--	--	1.27	-2.37	5.02	5.0%	--	--	--	--
active encouragement	0.07	0.07	-1.82	3.36	--	--	-0.12	-1.58	0.27	-0.5%	--	--	--	--
parental demand	**0.17**	0.06	**16.98**	3.98	**23.47**	3.52	**2.89**	**0.71**	**6.48**	11.5%	**5.42**	**2.64**	**10.86**	23.6%
parental allowing	**-0.17**	0.07	4.16	3.81	--	--	-0.69	-2.45	0.29	-2.8%	--	--	--	--
parental facilitation	**0.33**	0.08	**7.08**	3.53	**12.16**	3.44	**2.33**	**0.09**	**5.56**	9.3%	**4.24**	**1.78**	**8.28**	18.5%
bringing to school	**0.39**	0.04	5.93	3.26	--	--	2.30	-0.08	5.52	9.2%	--	--	--	--
school availability	**0.20**	0.03	4.41	5.50	--	--	0.89	-0.97	3.99	3.5%	--	--	--	--
Total							**12.15**	**5.12**	**20.36**	48.3%	**12.9**	**8.40**	**20.1**	56.3%

**Vegetable intake (n = 1356)**	total effect = **-12.5** (-21.4; -3.63)						direct effect model 1 = **-12.7** (-21.5; -3.86)				Direct effect model 2 = **-14.0 **(-22.7; -5.26)			

	path a		path b (model 1)		path b (model 2)		mediated effect (a*b) in model 1				mediated effect (a*b) in model 2			
	coeff	SE	coeff	SE	coeff	SE	coeff	95%CI^1^		proportion	coeff	95%CI^1^		proportion
knowledge	-0.03	0.02	9.65	5.39	--	--	-0.31	-1.47	0.11	2.4%	--	--	--	--
liking	-0.06	0.06	**5.19**	2.23	--	--	-0.31	-1.39	0.19	2.5%	--	--	--	--
active encouragement	-0.02	0.07	1.25	1.85	--	--	-0.03	-0.68	0.17	0.2%	--	--	--	--
parental demand	**0.11**	0.06	**7.29**	2.49	**10.2**	2.18	0.82	0.04	2.22	-6.6%	**1.23**	0.15	2.81	-9.7%
parental allowing	**-0.14**	0.06	1.49	2.22	--	--	-0.21	-1.09	0.35	1.7%	--	--	--	--
parental facilitation	0.00	0.06	3.32	2.19	--	--	0.00	-0.65	0.53	0.0%	--	--	--	--
bringing to school	0.02	0.02	**17.75**	6.64	**--**	--	0.30	-0.29	1.41	-2.4%				
school availability	0.01	0.01	-19.61	21.62	--	--	-0.11	-0.60	0.09	0.9%	--	--	--	--
total							0.16	-1.85	2.39	-1.3%	**1.23**	0.15	2.81	-9.7%

^1 ^bias-corrected 95% confidence intervals retrieved from resampling (n = 5000)

Model 1 - model including all potential mediators

Model 2 - model including only the significant mediators from model 1

All models are adjusted for sex, age, ethnic background and maternal educational level

**Bold **- significant estimates at the p = 0.05 level

That the 2009 sample had a lower vegetable intake than the 2003 sample could not be completely explained by the assessed potential mediators, since the direct effect (path c') was still significant after the potential mediators were taken into account. Results from the mediation analyses indicated that parental demand acted as a suppressor in the relationship between cohort and vegetable intake, meaning that the association between cohort and vegetable intake became stronger after the 'demand' variable was taken into account. The variables with a negative association with the cohort variable (knowledge of the Dutch vegetable intake recommendation, liking, active parental encouragement, parental allowing) did not show significant mediating effects.

## Discussion

The aim of this study was to assess differences in fruit and vegetable consumption and their potential determinants between two samples of 11-year-old schoolchildren in the Netherlands measured in 2003 and 2009. Overall, the results indicated that the mean fruit intake of the children measured in 2009 was higher than that of the children measured in 2003, independent of their gender, ethnicity and maternal level of education; however, vegetable intake was somewhat lower in 2009 than in 2003. Also more children from the 2009 cohort met the WHO recommendation. However, the observation that more children from the 2003 cohort met the Dutch recommendation for fruit intake may be a result of the different definitions. The Dutch recommendation states a minimum of two *portions or pieces of fruit*, whereas the WHO recommendation combines fruit and vegetable intake and uses total *grams per day*. Additional analyses revealed that the 2003 children reported a higher consumption of tangerines than did the 2009 cohort. Whereas one tangerine counted as one piece of fruit, in the calculations for grams per day it was considered to contribute 50 grams and most other popular fruits such as apples, pears and bananas were estimated to weigh 100 grams per piece. That the 2003 cohort ate more tangerines is in line with the season of measurement; in September and October these kinds of fruit are widely available in the Netherlands.

Regarding the presumed mediators, significantly more children in 2009 knew about the recommended fruit intake levels than in 2003, and some positive trends for other potential determinants of fruit and vegetable intakes were also found.

The finding that the schoolchildren in 2009 on average reported eating more fruit than in 2003 is encouraging. From the mediation analyses it appears that knowledge of the recommendation, parental demand and parental facilitation explains most of this difference. This may indicate that school programmes were indeed able to improve the children's knowledge of the recommendations for fruit intake and that this influenced their intake. It further suggests that the school programmes or other media activities were able to reach the parents, who subsequently changed their parenting practices regarding fruit intake. It is, however, surprising that liking or school availability could not explain the differences between the two samples. Most school-based programmes not only addressed the knowledge of the recommendations but also individual level determinants such as liking [18]. Results also show that liking was most strongly associated with fruit intake, but also that the level of liking did not significantly differ between the samples. This may indicate that even if the 2009 sample was more exposed to fruit-promoting school programmes, these programmes were not able to positively influence the liking for fruit. Conversely, the school availability significantly differed between the two samples but was not related to fruit intake. This might be because a very small proportion of children in the 2009 sample reported the positive availability of fruit at school.

Our results regarding fruit intake are somewhat similar to the very few earlier studies that are available for approximately the same period of time. Rasmussen *et al. *showed that in Denmark fruit intake improved between 2002 and 2006 among 11-, 13- and 15-year-olds; in all age groups among girls the proportion which state to eat fruit at least once per day improved from between 52% and 58% in 2002 to between 62.9 and 69.8% in 2006 [20]. The proportion of boys which state to eat fruit at least once per day improved from between 29.2 and 49.6% in 2002 to between 41.3 and 55.0% in 2006. Johnson and Hackett presented evidence for a positive trend in fruit and vegetable intake in Liverpool between 2000 and 2006 for 9-10-year-olds; the proportion of boys and girls reporting eating fruit on the previous day increased from 71.5% in 2000 to 76.8% in 2006 and from 70.7% in 2000 to 80.8% in 2006 respectively [28]. However, no other studies published on potential underlying factors explaining the trends in fruit intake.

The results further indicate that 11-year-olds in 2009 did not eat more vegetables than 11-years-olds in 2003. On the contrary, the 2009 sample reported a lower vegetable intake than the 2003 sample. This finding is consistent with results from previous research, indicating that increasing intake levels of vegetables of children is more difficult than improving fruit intakes. Children have higher preferences for fruit [29], and in the Netherlands it might be even more difficult to improve vegetable intake by school-based promotion, as vegetable intake during school hours is uncommon and does not fit the normal eating patterns of most native Dutch families. Vegetables are part of the evening meal, but are rather uncommon at breakfast, lunch or in between meals [16]. The potential mediators included in the current study could not explain the difference in vegetable intake between the two samples, but we found that parental demand and bringing vegetables to school had a suppressive effect on the difference in vegetable intake between the samples. This indicates that even though parental demand and bringing vegetables to school were significantly associated with the outcome variables in the expected direction, the so-called direct effect of the cohort variable on the outcome was stronger when these two variables were taken into account. These results suggest that there must be unmeasured factors that explain why the 2009 sample reported a lower vegetable intake than the 2003 sample. Therefore, future studies should include other potential mediators in order to inform future intervention strategies. One possibly important mediator may be feeding strategies, as a study by Zeinstra *et al. *showed that a feeding strategy in which children could make choice regarding when and what vegetables to eat, was positively associated with their vegetable intake [30]. In addition, since school availability of vegetables was very low, an alternative strategy may be to improve availability and accessibility of vegetables at schools, for example by providing ready-to-eat vegetables as a snack in the morning breaks.

The fact that many factors that have been found to be significant correlates of fruit and vegetable intakes appeared to be more favourable in 2009 than in 2003 is encouraging. The fact in particular that in 2009 children more often agreed that there are fruits and vegetables available at their school may indicate that schools have changed their policies or have participated in programmes that facilitate availability and accessibility, such as the Schoolgruiten project [16]. Analyses of differences in school policies between 2009 and 2003 will be conducted to gain more insight. Nevertheless, the proportion of children who agreed that fruit and vegetables were available at their school remained low in 2009 also, indicating that there is much room for further improvement.

There was one potential determinant that showed a negative trend: fewer children in 2009 than in 2003 reported that they were allowed to eat as much fruit and vegetables as they wanted. It may be that parents have become stricter in controlling their children's eating behaviours. It could also be a statistical artefact since in 2003 the scores were high and could only go down (regression to the mean), but this is mere speculation and should be further explored in additional research.

A strength of this study is that it used representative samples for the Netherlands, using the same validated methodologies [24], that were sensitive enough to detect changes in intake as well as in determinants [17]. Another strength of the study is that effect modification by gender, maternal educational level and ethnic background was explored and that potential confounders were controlled in multiple regression analyses. Moreover, the current study did not solely explore differences in intake; it also included potential determinants of fruit and vegetable intake and was thus able to conduct mediation analyses. There are also some limitations to this study, however. First, fruit and vegetable intake might have been influenced by seasonal effects, because the Pro Children data were collected in autumn and the Pro Greens data in spring. Since seasons may influence the availability of fruit and vegetables, it is generally assumed that children eat more fruit and vegetables in summer and autumn compared with winter and spring [31]. We explored this assumption in the available data from the control group of the Dutch Pro Children intervention study (n = 188) and indeed found that the children reported significantly higher intakes of fruit and vegetables in the September measurement compared with the May measurement (data not shown). The observation in the current study that the 2009 sample reported higher fruit intakes in May than the schoolchildren in October 2003 is not consistent with this proposed seasonal effect and may thus represent a real higher intake level in the 2009 sample compared with the 2003 sample. Furthermore, this seasonal influence is unlikely to apply to for most determinants, except for the availability of fruit and vegetables.

Second, the Pro Greens questionnaire slightly differed from the one used in Pro Children in the assessment of fruit intake, i.e. a separate question for berries was added. Although it was just a minor adaptation, a sensitivity analysis was conducted. There were doubts as to whether children might have thought that they had to state pieces of berries eaten instead of portions of berries eaten, because there were high intake numbers on this item. Therefore, under the assumption that the children reported pieces of berries instead of portions, the numbers were recalculated into portions eaten to check if it would make a difference in the number of children who reached the recommended intake. Results from these analyses showed that the reported differences between the two samples were not caused by this extra questionnaire item (data not shown).

A final limitation was the use of self-reported measures. Self-reported intake levels may be biased, but a previous study showed acceptable validity of the questionnaire used in the present study. Furthermore, any bias because of self reports is likely to be the same in the two study samples, and will therefore not be of limited influence on the comparison between the two samples.

Taking the limitations into account, the present study does provide an indication of the current situation compared with a period in which interventions and policies aimed at improving schoolchildren's fruit and vegetable intakes were not omnipresent. Although the effect size may not be clinically relevant for individuals, there is a likely relevance for public health, because many children can be reached by school-based interventions and small changes in a large proportion of the population can have an important impact on health indicators, as recently shown in an epidemiological modelling study [32]. In the Netherlands 608 out of around 7000 primary schools reported participating in Schoolgruiten or similar fruit and vegetable promoting projects. The result of the current study might indicate that the increased attention to fruit and vegetable intakes in Dutch schools may have started to have a somewhat positive effect among 11-year-olds.

## Conclusion

The outcome of the present study suggests that there was a small improvement in fruit consumption in 11-year-olds between 2003 and 2009, whereas the intake of vegetables was lower in the more recent sample. Children reported in general more knowledge of recommended intake levels and more favourable scores on other potential determinants of fruit and vegetable intakes in 2009 than in 2003. Improved knowledge, parental demand and parental facilitation explained the differences in fruit intake which suggests that these factors should be addressed in future intervention programmes or strategies.

## Competing interests

The authors declare that they have no competing interests.

## Authors' contributions

AY, JB, SJtV designed the study. CF, NIT and SJtV conducted the analyses. CF drafted the manuscript. All authors provided feedback on the manuscript and approved the submitted version.
